# Performance of an Ambulatory Dry-EEG Device for Auditory Closed-Loop Stimulation of Sleep Slow Oscillations in the Home Environment

**DOI:** 10.3389/fnhum.2018.00088

**Published:** 2018-03-08

**Authors:** Eden Debellemaniere, Stanislas Chambon, Clemence Pinaud, Valentin Thorey, David Dehaene, Damien Léger, Mounir Chennaoui, Pierrick J. Arnal, Mathieu N. Galtier

**Affiliations:** ^1^Rythm SAS, Paris, France; ^2^Unité Fatigue et Vigilance, Neurosciences et Contraintes Opérationnelles, Institut de Recherche Biomédicale des Armées, Brétigny-sur-Orge, France; ^3^EA7330 Vigilance Fatigue et Sommeil, Hôtel Dieu Paris, APHP, Université Paris Descartes, Paris, France; ^4^LTCI, Telecom ParisTech, Universitéaris-Saclay, Paris, France

**Keywords:** ambulatory sleep device, automatic sleep-staging, closed-loop stimulation, EEG wearable, sleep monitoring, sleep device, N3 sleep, slow-wave sleep

## Abstract

Recent research has shown that auditory closed-loop stimulation can enhance sleep slow oscillations (SO) to improve N3 sleep quality and cognition. Previous studies have been conducted in lab environments. The present study aimed to validate and assess the performance of a novel ambulatory wireless dry-EEG device (WDD), for auditory closed-loop stimulation of SO during N3 sleep at home. The performance of the WDD to detect N3 sleep automatically and to send auditory closed-loop stimulation on SO were tested on 20 young healthy subjects who slept with both the WDD and a miniaturized polysomnography (part 1) in both stimulated and sham nights within a double blind, randomized and crossover design. The effects of auditory closed-loop stimulation on delta power increase were assessed after one and 10 nights of stimulation on an observational pilot study in the home environment including 90 middle-aged subjects (part 2).The first part, aimed at assessing the quality of the WDD as compared to a polysomnograph, showed that the sensitivity and specificity to automatically detect N3 sleep in real-time were 0.70 and 0.90, respectively. The stimulation accuracy of the SO ascending-phase targeting was 45 ± 52°. The second part of the study, conducted in the home environment, showed that the stimulation protocol induced an increase of 43.9% of delta power in the 4 s window following the first stimulation (including evoked potentials and SO entrainment effect). The increase of SO response to auditory stimulation remained at the same level after 10 consecutive nights. The WDD shows good performances to automatically detect in real-time N3 sleep and to send auditory closed-loop stimulation on SO accurately. These stimulation increased the SO amplitude during N3 sleep without any adaptation effect after 10 consecutive nights. This tool provides new perspectives to figure out novel sleep EEG biomarkers in longitudinal studies and can be interesting to conduct broad studies on the effects of auditory stimulation during sleep.

## Introduction

Sleep is a complex process that plays a key role in maintaining homeostasis, well-being and overall health (Tononi and Cirelli, 2003; Besedovsky et al., 2012; Irish et al., 2015). In recent decades, increasing evidence has confirmed that slow-wave sleep (SWS) had a major impact in many biological functions such as glucose metabolism, hormone release, immunity, and memory (Van Cauter et al., 1997; Born, 2010; Xie et al., 2013; Varin et al., 2015; Besedovsky et al., 2017). This proposed role for SWS, coupled with observations of impaired SWS in several chronic pathologies such as fibromyalgia (Lentz et al., 1999), as well as in aging (Van Cauter et al., 1997; Scullin, 2012), have led to imagine the development of methods that could specifically enhance SWS (see Bellesi et al., 2014 for Review). Several pharmacological treatments, in which recently the GABA reuptake inhibitor Tiabagine has been tried to increase slow oscillations (SO) of SWS (Mathias et al., 2001; Walsh et al., 2006). Transcranial direct current stimulation and transcranial magnetic stimulation have also been shown to be able to induce slow waves (Marshall et al., 2006). However, since the long-term health effects of applying electrical stimulation to the brain are not yet known, more attention has been given to the possibility of enhancing slow waves by using less invasive means with “more physiological” stimuli. Among different sensory modalities, vestibular stimulation (Bayer et al., 2011) and auditory stimulation appeared to be effective in increasing the magnitude of SO (Ngo et al., 2013, 2015; Oudiette et al., 2013; Cox et al., 2014; Ong et al., 2016; Leminen et al., 2017; Papalambros et al., 2017).

However, given the few possibilities to analyze electroencephalography (EEG) sleep in real-time and the constraint imposed by closed-loop stimulation, these studies have been generally conducted in small groups of subjects, only in a laboratory environment and with only one night of polysomnography (PSG) with auditory stimulation in the desired stimulation condition after one habituation night (e.g., 11 participants in Ngo et al., 2013, 12 participants in Cox et al., 2014, 18 participants in the driving condition and 16 participants in the “2-Click” protocol of Ngo et al., 2015, 16 participants in Ong et al., 2016, 15 participants in Leminen et al., 2017, 13 participants in Papalambros et al., 2017). As raised by a recent study, this standard practice that involves EEG monitoring in appropriate sleep infrastructures requires important monetary, time and trained human resources costs for the development of the stimulation algorithm, the EEG hook-up, the overnight supervision, the triggering of the stimulation algorithm through the night, the EEG disconnection and the sleep scoring (Mihajlovic et al., 2015).

While numerous EEG devices have engaged in developing EEG solutions that can be used in daily life activities (Mihajlovic et al., 2015), fewer EEG devices have been specifically developed for sleep purposes trying both to file EEG recordings and to automatically sleep score (Van De Water et al., 2011). Among them, the now-discontinued Zeo device (Zeo, Inc., Newton, MA) seemed to be one of the most effective devices based on scientific performance assessments. The studies evaluating its performance concluded that the Zeo device was useful for sleep monitoring at home, with some weaknesses related to the over-scoring of REM sleep and the underestimation of wakefulness (Gumenyuk et al., 2011; Kudesia and Bianchi, 2012; Scullin, 2012; Shambroom et al., 2012; Tonetti et al., 2013; Honma et al., 2016).

To our knowledge, there are no integrated device on the market to analyse sleep EEG in real-time and also send auditory closed-loop stimulation on SO. The task is indeed complicated both from a hardware, software, ergonomics and algorithmic perspective. This also raises several experimental difficulties since there is no human monitoring the stimulation. For instance, one need a good enough characterization of sleep to insure that the subject is properly into deep sleep before stimulating and to stop the stimulation process whether a sleep change or any arousal or awakening occur.

The aims of our study were to assess (i) the performance of the Wireless Dreem Device (WDD) (in it's beta version) to detect N3 sleep automatically for auditory closed-loop stimulation on SO as compared to gold-standard miniaturized polysomnography (PSG) (part 1) and (ii) to test the effects of auditory closed-loop stimulation on brain response on a cohort with a higher number of subjects in an observational pilot study at home (part 2).

## Materials and methods

### Subjects and settings

#### Part 1: validation of the acquisition and accuracy performances of the WDD in a clinical study

Twenty-four healthy subjects were recruited and included in this clinical trial through local and university advertisements (9 women, mean age = 23.2 years, range 19–29 years, PSQI: 2.6 ±1.2, Beck: 1.3 ±1.8, HAD: 8.5 ±3.0). This experiment was performed by the Alertness, Fatigue, and Sleep Team (EA 7330) in the Hôtel Dieu Hospital. The local ethics committee approved the experimental protocol and complied with the tenets of the Declaration of Helsinki (Number of clinical trial: NCT02956161). All volunteers gave their informed written consent prior to participation. They received a monetary compensation for their time. Inclusion and exclusion criteria can be found in Table 1. Routine surveys and a medical interview with a physician ensured that they were non-smokers, had no history of neurological, psychiatric or endocrine disease, including any sleep disorder. All participants were free from medication except hormonal contraceptives. They were asked to follow a regular sleep/wake rhythm for at least 4 weeks prior to the experiment with 7–10 h per night and no daytime naps. Their sleep and wake patterns were assessed with a sleep agenda and a wrist-actimeter (Actiwatch TM; Cambridge Neurotechnology, Cambridge, UK) from 1 week prior to the beginning of the experiment through to the end of the protocol.

**Table 1:** Inclusion and exclusion criteria of part 1 experiment.

**INCLUSION CRITERIA**
healthy subject moderate morningness, intermediate or moderate eveningness chronotype (Horne & Östberg questionnaire)
**EXCLUSION CRITERIA**
sleep disorder according to the ICSD-3 or DSM-5 traveling away from more than a time zone in the previous month acute or chronic disorders (cardio-vascular, respiratory, neurologic, psychiatric) night shifts work smoking more than 5 cigarettes per day drinking more than 5 glass of alcohol per week consuming excessive drinks with xanthics (coffee, tea, coke more than 6 cups per day) having a body mass index >30kg.m−2 being pregnant
**ELIMINATION CRITERIA**
not filling the rules imposed by the study (light off, complying with wearing both devices) not completing the study


Subjects underwent four ambulatory home nights' monitoring with both the WDD and a PSG worn together. The PSG was set at the sleep laboratory between 5 and 8 p.m. Participants were asked to put on their headband and launch the recording prior to sleep by themselves. In the morning, they were asked to remove the electrodes and return the material to the sleep lab.

The first night consisted of a habituation night that was discarded from the analyses. The three following nights encompassed: (i) Sham condition where SO during N3 sleep was targeted but no sound was triggered (ii) Ascending condition where the ascending phase of the SO during N3 sleep was targeted with sounds triggered and (iii) Random condition where stimulation were randomly played on the ascending, descending, up and down phases of SO during N3 sleep. This study was double blind, randomized and crossover designed. The washout period was 1 week between each condition.

#### Part 2: neurophysiological responses of ERP components after auditory closed loop stimulation provided by the WDD in an observational pilot study

The study consisted of an observational pilot study on subjects who bought, consented for their data to be analyzed and published for research purposes and used the beta version of the Dreem headband ((Rythm sas, Paris, 2016), referred to as the WDD in the manuscript) from November 2016 to June 2017. The study was conducted in respect to the ethical standards of the Declaration of Helsinki. All servers, databases and services handling data were hosted on the secured Amazon WebServices. The entire infrastructure, with the exception of a few S3 storage buckets, were physically located in Frankfurt, Germany. All communications to and from the servers are strictly done via HTTPS. Each recording was associated to a specific user, identified by a unique anonymized identifier.

Since buying the device was a voluntary act, no exclusion criteria were followed except having a sleep or neurological disorder, as assessed by a questionnaire. Similarly, the number of nights spent with the headband was not controlled and the choice to wear the headband was left to the subject. There was no particular interaction with participants (except if they contacted us with questions on the use of the device). Both participants and customer service personnel who answered participants questions were unaware that nights with sham were performed. In this respect, the study was conducted in a near complete double blind setup.

**Figure 1:**
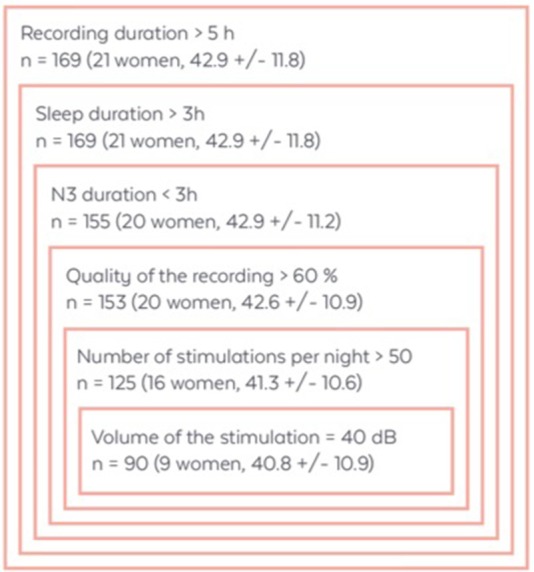
Demographic data of the total number of subjects after the application of each selection criteria. Data are presented as the number of subjects (n) with sex ratio and mean age ± SD in years.

To filter out inherent bad recordings due to the home environment which is controlled in a laboratory, recordings with a minimum duration of 5 h, a minimum effective sleep time of 3 h and a good EEG signal quality (higher than 60% of the time) were considered. To avoid the impact of outliers, recordings without N3 sleep or with more than 3 h of N3 sleep were removed from the analysis. Furthermore, we only kept subjects and recordings with more than 50 stimulation or sham by night and with a fixed volume of 40 dB to limit data heterogeneity (see Figure 1 for demographic data of the resulting population before and after applying the criteria of inclusion/exclusion).

### Materials

#### Polysomnographic recordings

The PSG device was comprised of miniaturized multi-channel ambulatory recording devices (Actiwave®, CamNtech Ltd England) with the following derivations: 6 EEG: Fp1-M2, C3-M2, O1-M2, Fp2-M1, C4-M1, O2-M1, 2 electro-oculograms (EOG), 2 chin electromyograms (EMG), and an electrocardiogram (ECG) (Sauvet et al., 2014). The habitual frontal derivations were replaced by Fronto Polar position (FP) in order to be placed directly adjacent to the headband electrodes position. Bio-electrical signals were digitized at a sampling frequency of 128 Hz with a 10-bit quantization between −500 and +500 μV, within a bandwidth of 0 to 48 Hz. All the data were stored in computer files using the standard .EDF data format. EEG cup-electrodes of silver- silver chroride (Ag-AgCl) were attached to the scalp of the subjects (EC2 electrode cream, Grass Technologies, An Astro-Med, West Warwick, USA), according to the international 10-20 system for electrodes placement. Auto-adhesive electrodes (Neuroline 720, Ambu A/S, Ballerup, Denmark) were used for EOG recordings. The recording devices were fixed on the subjects' heads using EC2.

#### Ambulatory Dry-EEG device: the WDD

**Figure 2:**
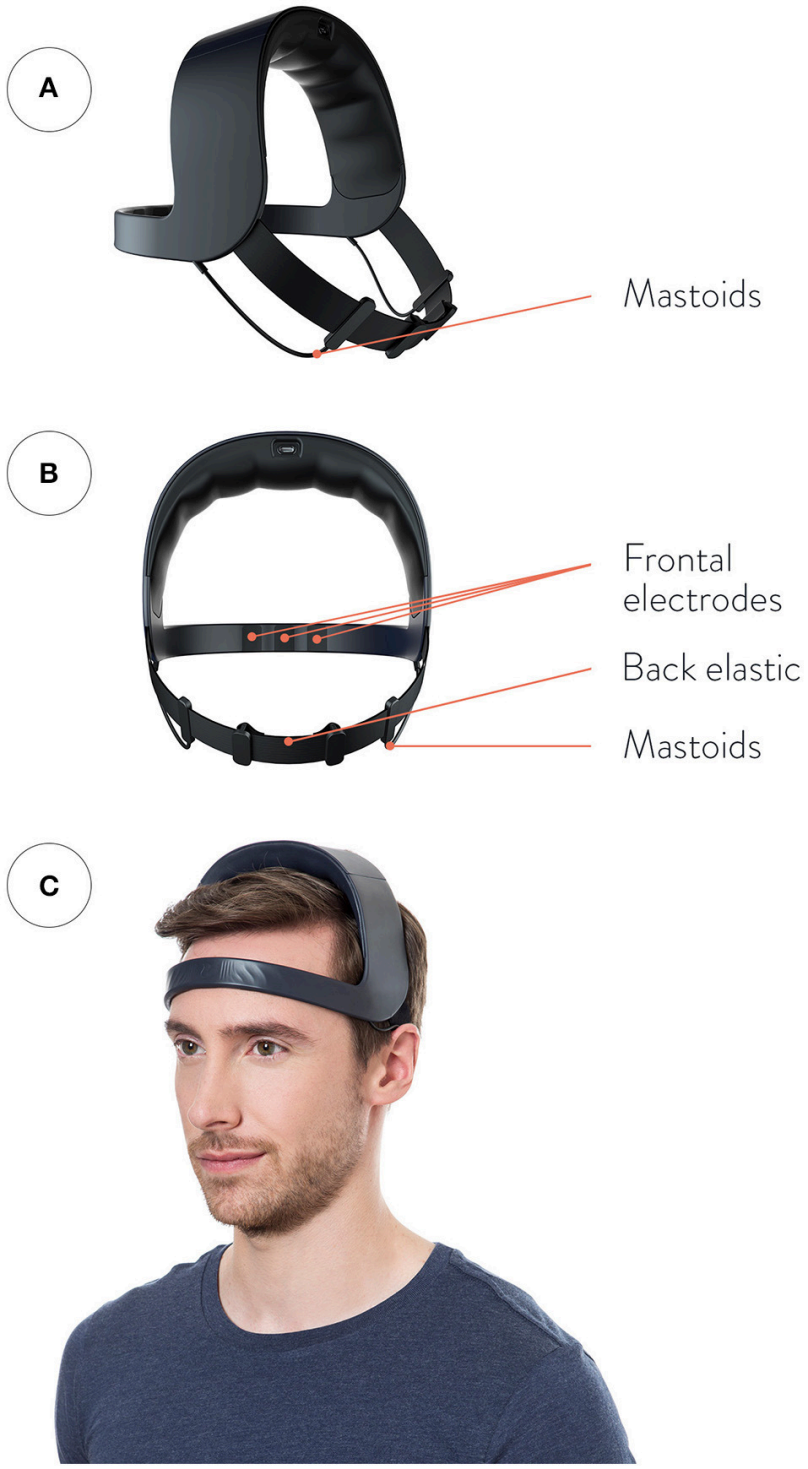
Representation of the WDD. **(A)** Front view, **(B)** Back view, **(C)** Side view. The device is made up of four dry measuring electrodes: two front sensors placed in Fp1, Fp2, and two “reference electrodes” placed behind the ears as “mastoids” electrodes. The top arch gathers all the electronic components. (Please note that the model gave his written informed consent for the publication of this image).

The WDD device is a wireless system using 5 dry nanocarbon-coated fabric sensors to record EEG signal in ambulatory. The 4 EEG derivations were FPp1-M1, Fp2-M2, Fp1-Fpz and Fp1-Fp2 where Fpz was the virtual ground (Figure 2). The 2 derivations used for sleep analysis were FPp1-M1 and Fp2-M2. Unconventionally, the derivations were not contra-lateral wired because unilateral derivations improve the signal quality of the WDD by limiting electrodes detachment artifacts. Indeed, when sleeping on their side, it is generally one entire side that is artifacted. The WDD is available in a unique size that fits all thanks to the elastic band behind the head that makes it adjustable such that it is tight enough to be secure, but loose enough to minimize discomfort. The signal is measured at 250 Hz, filtered in the 0.4–18 Hz band and post-processed according to the algorithms described below. An accelerometer is embedded in the WDD providing movements of the head at a sampling frequency of 50 Hz. A bone conduction device, integrated in the frontal band of the WDD on the forehead, delivers sounds. This minimizes the sound that travels through air while keeping a perceptive sound loud enough for the headband wearer. The noise level as compared to more traditional air conductor such as earphones was assessed in an external sound laboratory.

### Data analysis

#### Embedded real-time algorithms

**Figure 3:**
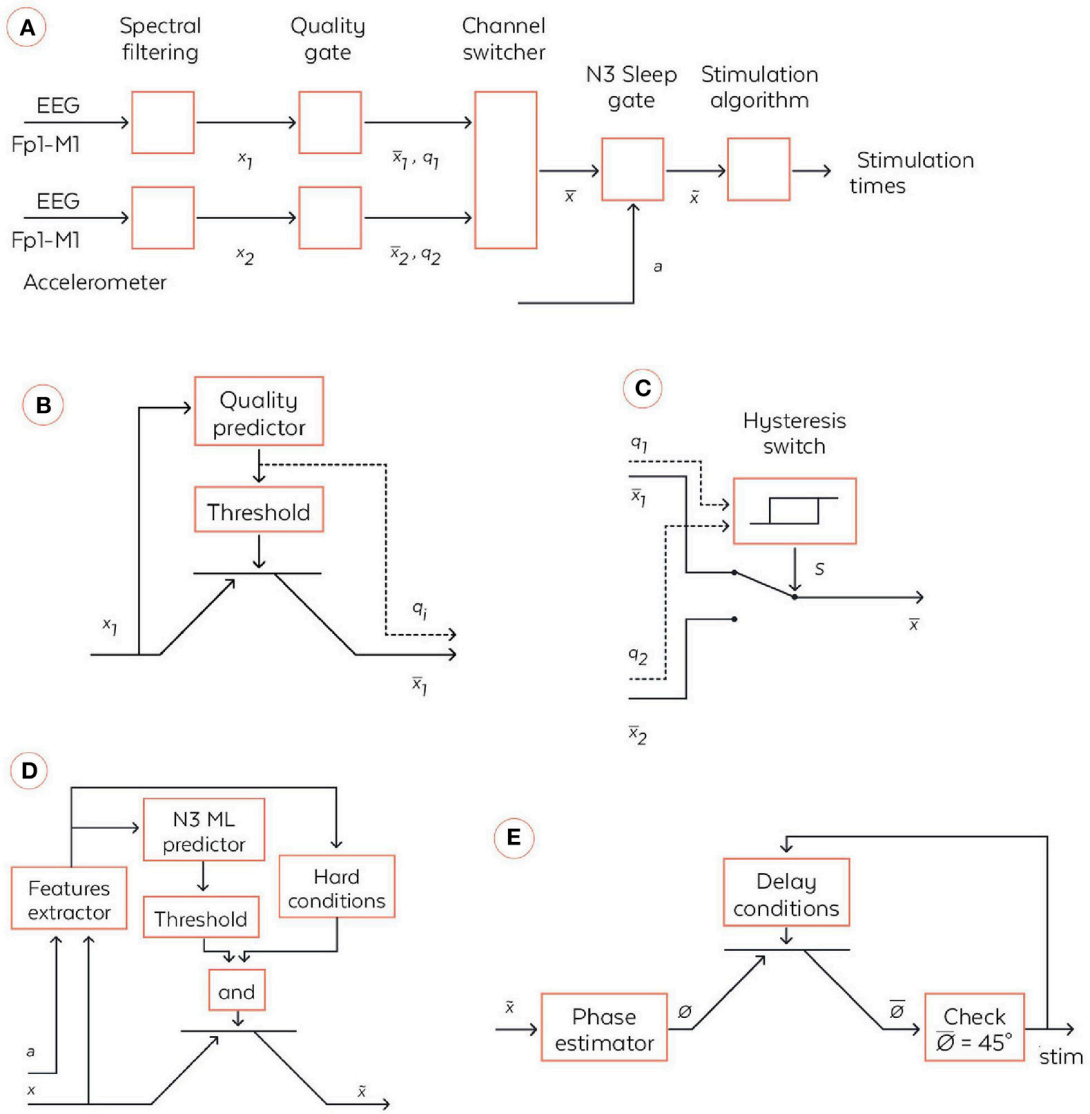
Coarse-grained algorithmic pipeline used by the WDD for stimulating N3 sleep. **(A)** General pipeline. **(B)** Quality gate representation, High-level block diagram of the quality gate. The filtered signal x_i_ (with i∈{1,2}) are sent to the quality predictor which computes an index of the signal quality q_i_∈[0,1] (0 means bad signal and 1 means perfect signal). This is a quantification of the extent to which the signal is perturbed by external artifacts, e.g., due to dry electrode bad contact. This quality index is the compared to a threshold to open or not the quality gate, which is illustratively represented via a transistor symbol. The output signal ${\bar{x}}_{\text{I}}$ is equal to x_i_ if q_i_ > {threshold} and is Not a Number (NaN) else. **(C)** High-level block diagram of the channel switcher. The two quality indices q_1_ and q_2_ enter an hysteris switch which selects which inputs, ${\bar{x}}_{1}$ or ${\bar{x}}_{2}$, are broadcasted to the next block. The hysteris switch is parameterized by a threshold θ and outputs a binary variable at time t computed as s(t) = 1 if q_1_-q_2_ > θ or [–θ < q_1_-q_2_ < θ and s(t-1) = 1]. Symmetrically, s(t) = 0 if q_1_-q_2_ < –θ or [–θ < q_1_-q_2_ < θ and s(t-1) = 0]. If s(t) = 1 then $\bar{x}$ = ${\bar{x}}_{1}$ and, if s(t) = 0 then $\bar{x}=$ ${\bar{x}}_{2}$. **(D)** N3 Sleep gate representation. High-level block diagram of the N3 sleep gate. Both inputs, the accelerometer a∈ℝ^3^and the virtual channel $\bar{x}\in \mathbb{R}$, are used in a series of operations to identify whether, the input corresponds to N3 sleep, in which case the virtual channel is broadcasted to the next block. More precisely, a and $\bar{x}$ enters a series of signal processing functions in the feature extractor. Not only are basic statistics of the two signals computed, but also some classical patterns of N3 sleep such as spindles and SO are identified. The extracted features are sent to a machine learning predictor which outputs an estimation of the probability to be in N3. In parallel, the features are used together with time information to check if several hard conditions are met: once the first N3 is detected, wait 15 min before starting to; do not stimulate if a large movement happened less than 3 min ago; stop to stimulate 4 h after the first detection of N3. If both the hard conditions are met and the N3 machine learning predictor outputs a probability to be in N3 larger than a threshold then $\tilde{x}=\bar{x}$, else $\tilde{x}=NaN$. **(E)** High-level block diagram of stimulation algorithm. First, the virtual channel is sent to a block, which estimates the phase of the signal in the delta band. We used a new phase detection algorithm here, which is described in the text body. Next the algorithm checks whether the phase is equal to 45°, the target that is set for stimulation, and emits a stimulation. However, there is a delay condition from the previous stimulation to ensure we do not stimulate at each SO. The two rules used here are: (i) do not stimulate more than two SO in a row, (ii) wait at least 9 s before the previous pair of stimulations. If those delays conditions are met, then the stimulation order is given to the hardware, which emits 50 ms stimulation through the bone conduction.

To produce auditory stimulation at a precise moment, the WDD implements a complex pipeline of operations, which is presented in a simplified form and detailed block-by-block below (Figures 3A–D). Overall, the three inputs of the pipeline are the two frontal-mastoid EEG derivations x_1_ and x_2_ and the three-dimensional accelerometer variable denoted a. Both EEG channels are filtered a priori with a combination of infinite impulse response filters (Figure 3A). More precisely, the signals were filtered with the following causal filters: a 4th order bandpass butterworth filter in the 0.4–18 Hz frequency band (to restrict to meaningful frequencies in sleep analysis), a 58–62 Hz bandstop 6th order butterworth filter (to remove the North American power line), a 48–52 Hz bandstop 6th order butterworth filter (to remove the European power line) and a 62–63 Hz bandstop 2nd order Bessel filter (to remove frequencies which are generated by the headband for calibration).

##### Quality gate

The quality gate allows the signal to proceed to the next stage if it reaches a threshold quality (Figure 3B). This quality detector is a machine learning predictor (forest of decision trees) applied to a binary classification task on a large database of 2 s windows labeled by sleep experts which specified if parts of the signal correspond to good or bad quality signal. Every 0.5 s, an estimation of the quality is made by this algorithm, returning a number between 0 and 1. If it crosses the minimal threshold then the signal is broadcast to the channel switcher. The accuracy of this detector and its AUC ROC were of 0.967 and 0.982, respectively, on the testing dataset.

##### Channel switcher

The algorithm selects the channel with the highest quality (Figure 3C). This so-called selected channel is referred to as the “virtual channel.” A hysteresis switcher avoids switching too often from one channel to the other if they have similar quality.

##### N3 sleep gate

The N3 sleep gate classifies 30 s windows of “virtual channel” in N3 sleep *vs* the other sleep stages (referred to as “else”) (Figure 3D). This N3 sleep detector is mainly comprised of a machine learning predictor (forest of decision trees) fed with numerous features computed on the “virtual channel” and on the accelerometer Figure 3E. For instance, we considered relative power in frequency bands on the EEG signal (estimated with spectral density) in intervals 0.4–4 Hz (for the delta band), 4–8 Hz (for the alpha band), 8–12 Hz (for the sigma band), and 12–18 Hz (for the beta band), permutation entropy of EEG and various measures of signal complexity to distinguish N3 from else. We also identify key sleep patterns in the signal such as spindles and slow oscillations. If the signal is detected as N3 sleep and meets hard conditions applied to avoid awaking the user, then it is broadcast to the next stage. In other sleep stages, the data are not sent to the stimulation stage and no stimulation can be heard. Notably, the WDD does not stimulate if the quality of both channels is bad.

##### Phase fitting algorithm

**Figure 4:**
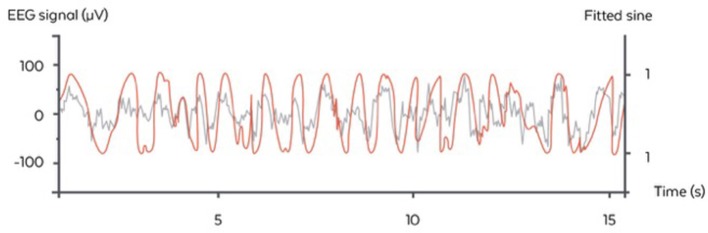
Illustration of the stimulation algorithm on a 15 s epoch of EEG during N3 (orange). At each time, a sinus with the appropriate frequency is fitted to the last few seconds of the signal. Here, only the current value of the sinus is displayed (gray) and serves as a basis for stimulation in the ascending phase. One stimulation trigger is shown in red.

The algorithm used here was inspired by the Cox et al. and consisted of fitting a sinus to the filtered 0.4–4 Hz signal of the “virtual channel” (corresponding to the delta range frequency of interest) and identifying the phase of the signal on the sinus itself (Cox et al., 2014). In the considered case of a fixed frequency, this fit corresponded to a linear regression performed in real time and at each time step with a recursive least square method with a forgetting factor of λ = 0.99 providing a “memory” (the equivalent of the size of a sliding window) of 5 s. The fit was performed for 5 regularly spaced frequencies for the sinus between 0.8 and 1.2 Hz, i.e., freq_list_ = [0.8, 0.9, 1, 1.1, 1.2]. The frequency with the best fit was chosen. At each time step, another sinus was fitted to the signal and only the last value was considered for stimulation. We used a Recursive Least Square method to perform the fitting economically at each time step (Adali and Haykin, 2010) The frequency with the best fit was chosen. At each time step another sinus was fitted to the signal and only the last value was considered for stimulation. This resulted in a phase approximation, which is different than a normal sinus (Figure 4). The algorithm in pseudo code with the following notations:

− t is a column vector of regularly timed steps between 0 and 2 s at a sampling frequency with *n* = 250 rows
− Λ is a diagonal matrix with diagonal (1, λ, λ^1^, λ^2^,… λ^(n−1).^).

Initialization

> for f in freq_list_:

$$\begin{array}{lll} D_{f}\; & = & \;\left[\cos\left(2\;*\;\pi \;*\;f\;*\;t\right),\sin\left(2\;*\;\pi *f\;*\;ti\right)\right]\\ V_{f}\; & = & \;D_{f}.T\;*\;\Lambda^{1/2}\;1/2\;*\;Df^{-1}\\ W_{f}\; & = & \;Vf\;*\;Df.T\;*\;\Lambda^{1/2} \end{array}$$

At each time step, for a new signal y

> for f in freq_list_:

$$\begin{array}{lll} \theta_{f} & = & \;W_{f}\;*\;y\\ \varphi_{f} & = & \;-\;\arctan\left(\theta_{f}\left[0\right],\;\theta_{f}\left[1\right]\right)\\ correlation & = & \;\left(y.T\;*\;D_{f}\;*\;\theta_{f}\right)\;/\;\left(y.T\;*\;\Lambda \;*\;y\right) \end{array}$$

> Choose f and φ_*f*_ that maximize the correlation.

##### Stimulation procedure

The stimulation was launched based on the estimated phase according to the previous procedure. In part 1, the acoustic stimulation routine was inspired from previous studies and consisted of two consecutive SO phase-locked stimulation of 40 dB pink noise (Ngo et al., 2015; Ong et al., 2016). The exact phase targeting was 45° in the ascending condition, just before the up-phase, since after some piloting, it seemed to be the optimal phase for driving SO. Part 2 included only nights with stimulation (i.e., no nights with sham only) since WDD users almost systematically turned on the stimulation when using the headband. However, in these so-called “stimulation nights,” approximately 50% of the stimulations were real stimulations (i.e. with sounds set at 40 dB) and approximately 50% of the stimulations were sham (i.e., with no sound). Sham and real stimulations were randomly displayed through the night.In both studies, a pause of 9 s, minimum, between trains of two stimulations was made before detecting another SO and stimulating. Stimulations began after 15 min of stable N3 sleep and persisted during this sleep stage solely, unless a movement or alpha rhythm was detected in the 6 s following the stimulation. In which case, a 30 s pause was initiated. The choice of waiting 15 min before stimulating ensured we did not wake the subject.

#### A posteriori data analysis

##### Resynchronization procedure

In part 1, a resynchronization procedure was processed between the EEG signals provided by the PSG device and the WDD. Indeed, a time lag exists between the signals because each device has its own clock. The clock have different precisions and they are not synchronized. Moreover, external factor such as temperature may affect the sampling frequency along the night. This results in non-linear and non-monotonous time lag along the night between both signals. The order of magnitude of this time lag is such that it can represent seconds at the end of an 8 h long record. Thus, a sequential resynchronization procedure for chunks of 10 min of recording was used where the problem was expressed as an optimization problem as a function of signal translation and sampling frequency to solve the time lag.

##### Signals correlation methods

Correlation between PSG and the WDD was assessed on resynchronized signals with a Pearson correlation coefficient for windows of 2 s. Signals with detached electrodes were removed from the analysis (1.19% of the signal removed because of two bad PSG derivations, 4.72% because of two bad WDD derivations, 10.07% because of one bad PSG derivation and 13.11% because of one bad WDD derivation). The correlation between the PSG and the WDD could not be computed on the same derivations since the wiring of the WDD is unilateral (Fp1-M1, Fp2-M2). The classical wiring of the PSG was not changed into unilateral montage to avoid impairing the sleep stage classification of sleep experts who are used to a contro-lateral montage. Therefore, we compared the 'virtual channel' of both the WDD and the PSG. Eventually, the pair of channels that were compared always had a common location for one electrode, to the extent that both devices have to be set up to slightly different locations. Overall, this imperfect “virtual channel” comparison underestimated the results of correlation and served as a lower bound to the real correlation.

##### Performance analysis of automatic N3 sleep detection

The performance analysis of the automatic N3 sleep detection of the WDD was assessed on the recordings from Part 1 by comparing the performance of the device to the manual sleep scoring of an expert on the PSG. The trained research technician was blinded to the conditions and scored the signals in accordance with AASM criteria (Iber et al., 2007) using SOMNOLOGICA (TM; Medcare, Reykjavik, Iceland). Stages, annotations and timestamps were recorded. Note that none of the night analyzed here was involved in the training of the embedded automatic sleep staging algorithm. To assess the performance of the WDD, we determined the true positive rate (i.e., correct N3 sleep detected by the WDD), false positive (i.e., false N3 sleep detected), true negative (i.e., correct N3 sleep rejected), false negative (i.e., false N3 sleep rejected), sensitivity (i.e., correct N3 sleep detection when the PSG also scores SWS) and specificity (i.e., ability of the WDD to measure false N3 correctly identified as such).

##### Accuracy of the stimulation

The ability of the algorithm to target the positive half-wave (i.e., the ascending phase) of the SO was tested on the recordings from the clinical study (Part 1) to ensure that only stimulation elicited in N3 were analyzed. All the stimulations were summed up in a circular “polar plot” histogram by using a zero-phase digital filter with transfer function coefficients of a second order band-pass Butterworth filter in the delta band (0.4–4 Hz). The phase angle at each pulse delivery was identified and a Hilbert transformed was applied on the EEG signal to identify the instantaneous phase at each pulse delivery. Circular histograms were created with 72 bins of 5° where 90° represents the peak of the upstate and the ascending targeted phase of stimulation delivery 45°.

##### Event-related potentials

The impact of the stimulation on the EEG was assessed on the recordings from Part 2 in order to increase the statistical power by the important size of our sample (90 subjects; 10,512 stimulations and 9,872 sham triggers).

The averaged event-related potentials (ERP) are presented as mean ± standard deviation. In order to avoid phase delays, the spectral filtering of the signals was done with the same filters as previously described, but with a non-causal forward/backward scheme. The ERPs were time locked to both first and second trigger since, as opposed to former algorithms, the duration between two stimulations could vary due to the particular shape of the signal. This method choice implies some non-causality in the filtered signals (i.e., may lead to significant difference prior the first stimulation) but guarantees no phase delay.

#### Statistical analysis

**Figure 5:**
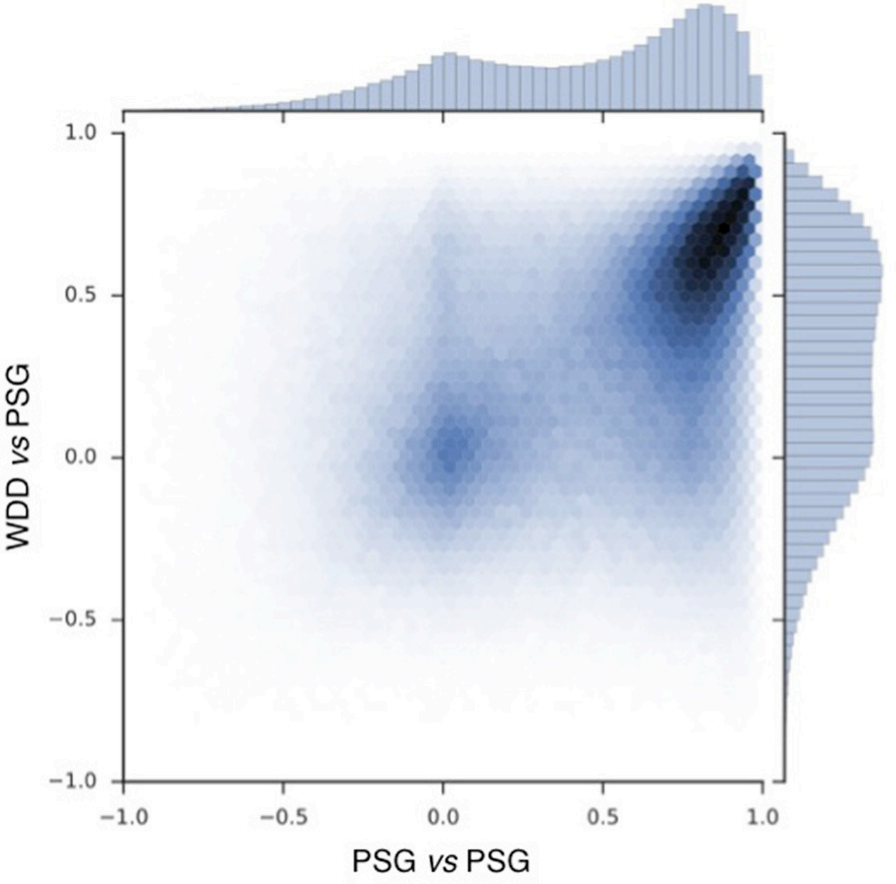
Pearson correlation scatter plot for 697017 windows of 2 s with resynchronized PSG and the WDD recordings. PSG vs. PSG shows the correlation between the two frontal channels of the PSG device. PSG vs. the WDD shows the correlation between the virtual channels of the WDD and PSG channel.

To compare the signals recorded by the WDD to the one recorded by the PSG, a Pearson correlation was made in each 2 s window (Figure 5).

In order to assess the impact of the stimulation on the ERP component depending on the condition, the sham (*n* = 9,872) and the stimulation (*n* = 10,512) triggers relative to each subject were averaged in reference to the first (**Figure 10A**) and second trigger (**Figure 10B**). A paired *T*-test was then applied to account for individual differences. A *p* < 0.001 was considered as statistically significant.

The increase in the delta band was computed between stimulated and non-stimulated SO. More precisely, we computed the delta power in the 0.4–4 Hz frequency band in a 4 s window following the first stimulation (or sham) in each train of 2 stimulations (or shams) (**Figures 11A,B**) and in a 4 s window following the end of the second succeeding the second and last trigger (**Figures 11A,C**). We used the squared norm of the discrete Fourier Transform of the 1,024 time steps after the first trigger convolved with a Hann function. This provided two distributions of delta power: one after stimulation and one after sham. We then computed the percentage of increase between the mean of these two distributions. We used a Paired *T*-test for the mean of the two independent distributions: the average delta power after stimulation (rest. Sham) was computed for each user. Then the two 90 points distributions were compared with a paired *T*-test, which involves linking the data points coming from the same uses. A threshold of *p* < 0.001 was considered to establish a significant difference between the two distributions. The dispersion of the subjects depending on the delta power increase of the stimulation as compared to the sham triggers (Δ Delta power) was calculated as follows:

$$\left[\left(\frac{Delta\;power\;Stim}{Delta\;power\;Sham}-1\right)\right]\times 100$$

(**Figures 11B,C**).

To measure the extent of habituation to stimulation, we compared the ERP after one and 10 consecutive night of stimulation in 24 subjects (**Figure 12**).

For each subject, the difference between the ERPs, time locked to the first trigger, of the averaged sham and stim conditions was computed after the 1st (Night 1) and the 10th night (Night 10). A paired *T*-test was then applied to assess for statistical difference between these two nights. A *p* < 0.001 was considered as statistically significant.

Statistical analysis were made using the SciPy library of Python.

## Results

### Part 1-validation of the acquisition and accuracy performances of the WDD in a clinical study

#### Data characteristics

In part 1, data from four participants were discarded from the analysis: one due to defective headband, one due to damaged PSG, one due to poor sleep of the participant among the four nights which might be explained by the discomfort of the set-up and one due to the disrespect of the protocol regarding sleeping hours. This resulted of a final sample size of 20 subjects (7 women, mean age = 23.1 years, range 19–29 years, PSQI: 2.6 ± 2.1, Beck: 1.2 ± 2.0, HAD: 8.6 ± 3.2) and 60 nights corresponding to the three conditions: Sham, Ascending and Random. The final sample did not statistically differ from the excluded subjects data in any criteria (age, sex, PSQI, Beck, and HAD).

#### Signal quality

Correlation scatter plot shows how two PSG channels correlate and how the WDD correlates to PSG (Figure 5). The Pearson correlation between WDD and PSG signal show a maximum around the value of 0.6.

**Figure 6:**
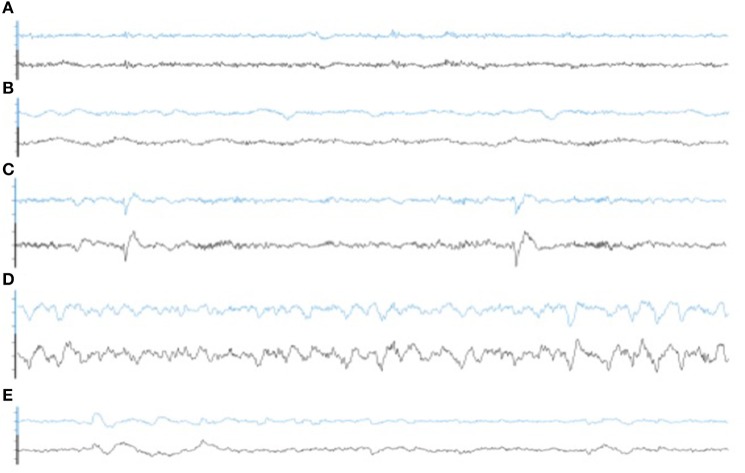
Representative 30 s epoch of **(A)** Wakefulness, **(B)** N1, **(C)** N2, **(D)** N3, **(E)** REM obtained with the simultaneous recording of the WDD (blue) and the PSG (black).

**Figure 7:**
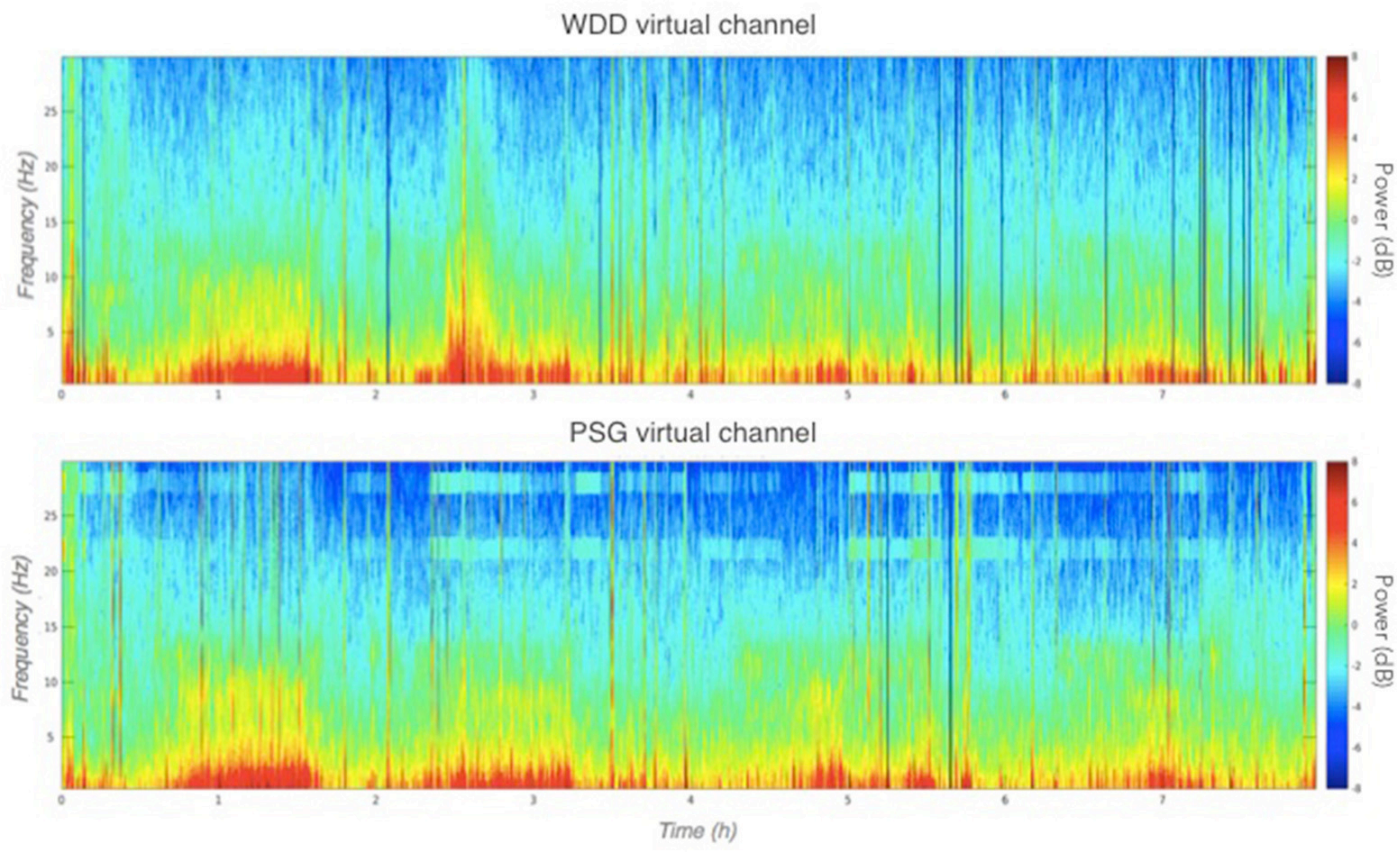
Representative multitaper EEG spectrogram of a full sleep night from the WDD (**Top**) and on the PSG (**Bottom**) recordings.

Illustrative samples of the signal obtained with the PSG and the WDD for each sleep stage are presented in Figure 6. The typical rhythms, including alpha and theta as well as the typical sleep patterns such as spindles, K-complexes and SO, were distinguishable in both recordings. Time-frequency plots show very similar distribution of frequencies across the night when comparing the two devices (Figure 7 for a representative plot, see all individual plots in Figure S1).

#### Automatic N3 detection

The performance of WDD to automatically detect N3 sleep with an algorithm as compared to the traditional sleep staging provided by the sleep expert on the PSG show high specificity (0.90), compared to sensitivity (0.70). Out of 42,302 total epochs scored, 12,276 epochs were scored in N3 with 3,017 epochs appearing as false positive, 3,666 as false negative, 8,610 as true positive and 27,009 as true negative (Table 2).

**Table 2:** Performance of the Wdd's automatic sleep stage algorithm.

**FP**	**FN**	**TP**	**TN**	**Sens**.	**Spec**.	**Prec**.	**Acc**.
3,017	3,666	8,610	27,009	0.70	0.90	0.74	0.84

*FP, False Positive; FN, False Negative; TP, True Positive; TN, True Negative; Sens., Sensitivity; Spec., Specificity; Prec., Precision, Acc., Accuracy*.

**Figure 8:**
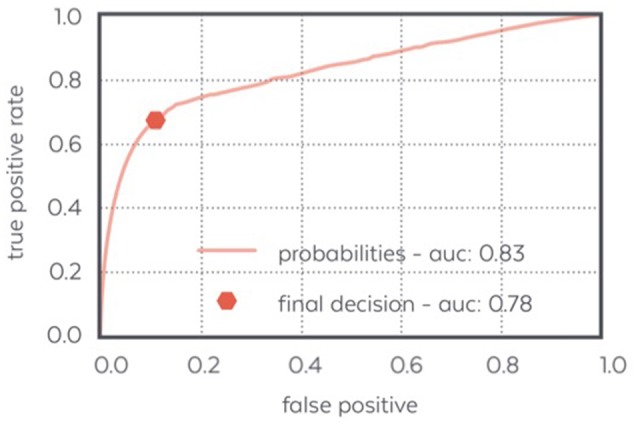
ROC curve of the N3 detector: the final decision made by the detector achieves a low false positive ratio and a quite high positive ratio which makes it able to detect most of the N3 samples confidently.

Figure 8 displays the ROC curve characterizing the algorithm performance and illustrating our decision to design an algorithm with few false positive.

A total of 17,579 stimulations were elicited by the device and 17,786 Sham. We observed that 86.1% of stimulation or sham were elicited in N3, 11.0% in N2, 0.4% in N1, 1.4% in REM and 1.1% during wakefulness according to the sleep expert (Table 3. See Figure S2 for the individual hypnograms and the stimulations triggers). The relatively high number of 13.9% of stimulations out of N3 resulted in both artifacts which were wrongly classified by the algorithms, which generated multiple spurious stimulations and to the fact that every 30 s epochs following an epoch scored as N3 will be stimulated, because the aglorithm only updates at the end of each epoch.

**Table 3:** Number of stimulations by sleep stage according to the sleep expert scoring.

	**Wake**	**N1**	**N2**	**N3**	**REM**	**NS**	**Total**
SHAM	122	68	1,760	15,343	282	4	17,579
STIM	267	70	2,126	15,112	197	14	17,786

*NS, Not scorable by the expert*.

#### Stimulation accuracy

**Figure 9:**
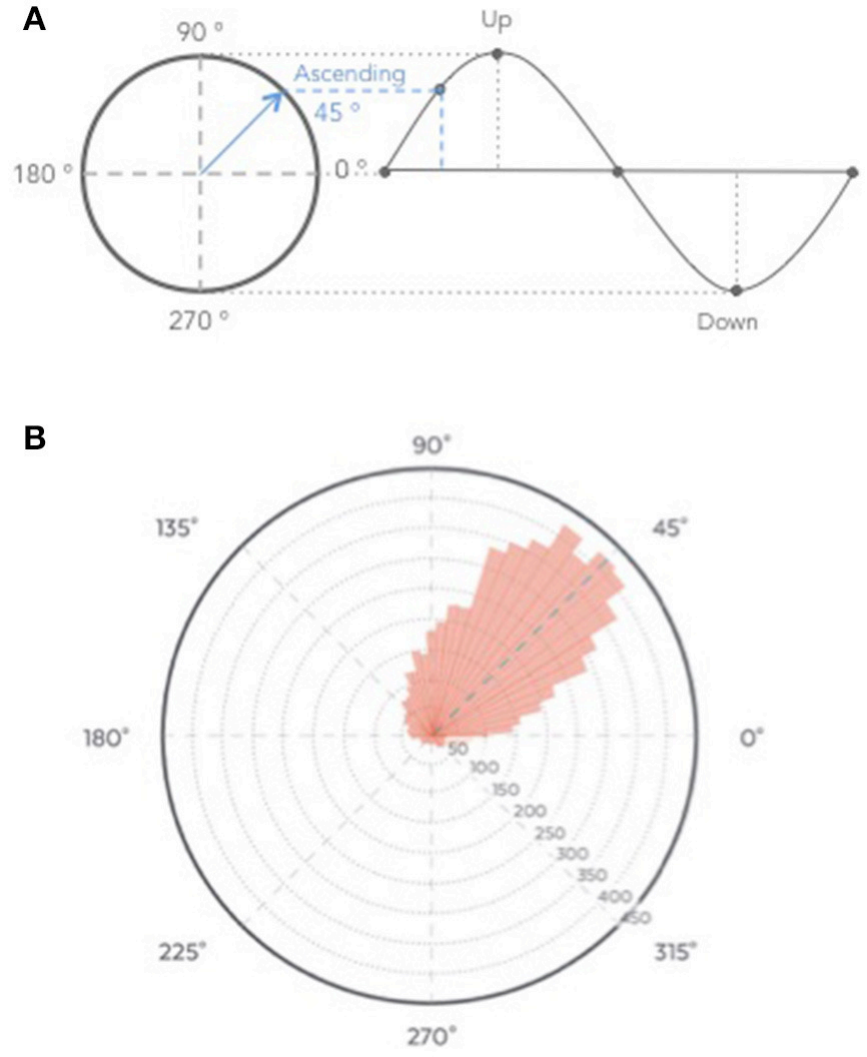
**(A)** Graphical description of phase angle. **(B)** Polar histogram showing 7,059 stimulations as a function of the phase of the signal. The targeted phase was 45° which represents the middle of the ascending slope. 90° corresponds to the peak of the up state, 270 degrees to the trough of the down state **(B)**.

The average time of stimulation for the 7,059 pink noise at the ascending phase was 45 ± 52° (Figure 9, see Figure S3 for individual polar plot).

### Part 2-neurophysiological responses of ERP components after auditory closed loop stimulation provided by the WDD in an observational pilot study

#### Data characteristics

In part 2, after applying the selection criteria to ensure the quality of the nights (see Material and Methods), 90 subjects (9 women, mean age: 40.8 ± 10.9 years old) were included for ERP impact of auditory stimulation. The longitudinal analysis involving 10 nights of stimulation in a raw only included 28 subjects (1 woman, mean age: 45.4 ± 8.0 years old). Indeed, since this study was observational, subjects were not asked to repetitively wear the WDD and most of them wore it sparsely (a couple days a week).

#### Neurophysiological impact of the auditory stimulation

**Figure 10:**
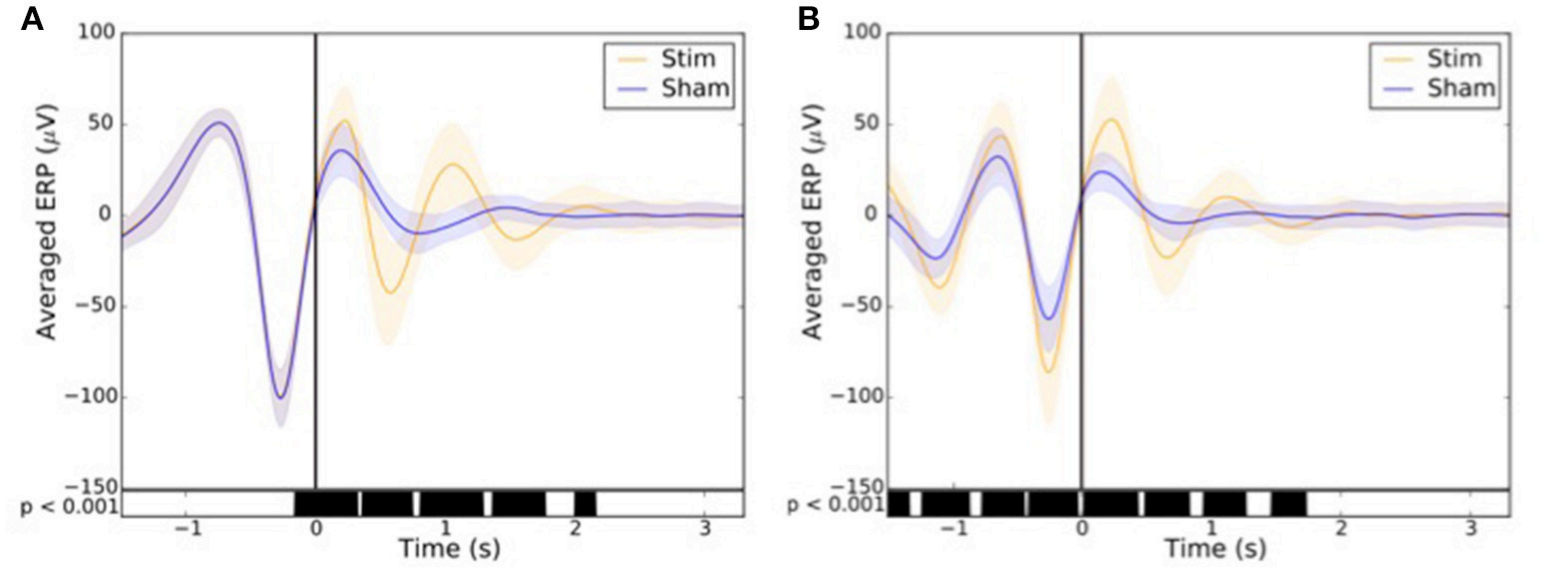
Averaged ERP (±SD) time locked to the first **(A)** and second **(B)** stimulus for the stim (orange line) and sham (blue line) in the observational study (Study 2). Black bars indicate time points where differences between the two conditions were statistically significant (*p* < 0.001).

**Figure 11:**
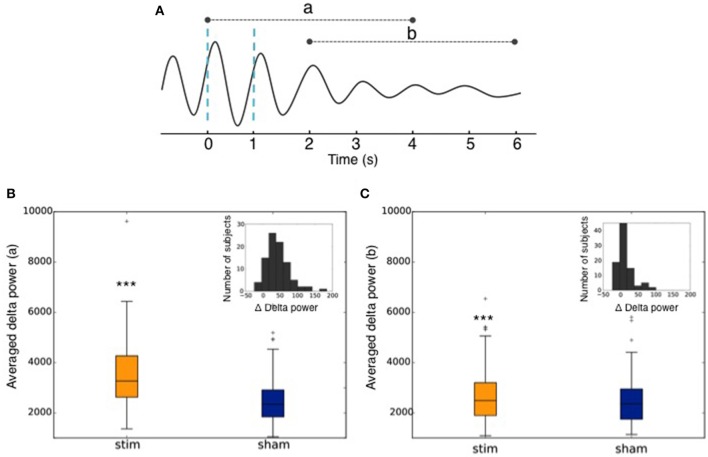
**(A)** Graphical representation of the averaged ERP used to calculate the delta power increase (a) of the 4 s following the 1st trigger, and (b) of the 4 s following the end of the second after the second trigger. Dotted blue lines represent stimulations. **(B)** Averaged power in the delta band in the 4 s following the 1st stimulation (Stim) or sham trigger (Sham). The repartition of the number of subjects depending on the delta power increase of the stimulations as compared to sham (Δ Delta power) is shown in the upper right corner. **(C)** Averaged power in the delta band in the 4 s following the end of the second after the second trigger (Stim) or sham trigger (Sham). The repartition of the number of subjects depending on the delta power increase of the stimulations as compared to sham (Δ Delta power) is shown in the upper right corner. ^***^ indicates significant difference between the Stim and the Sham condition (*p* < 0.001).

**Figure 12:**
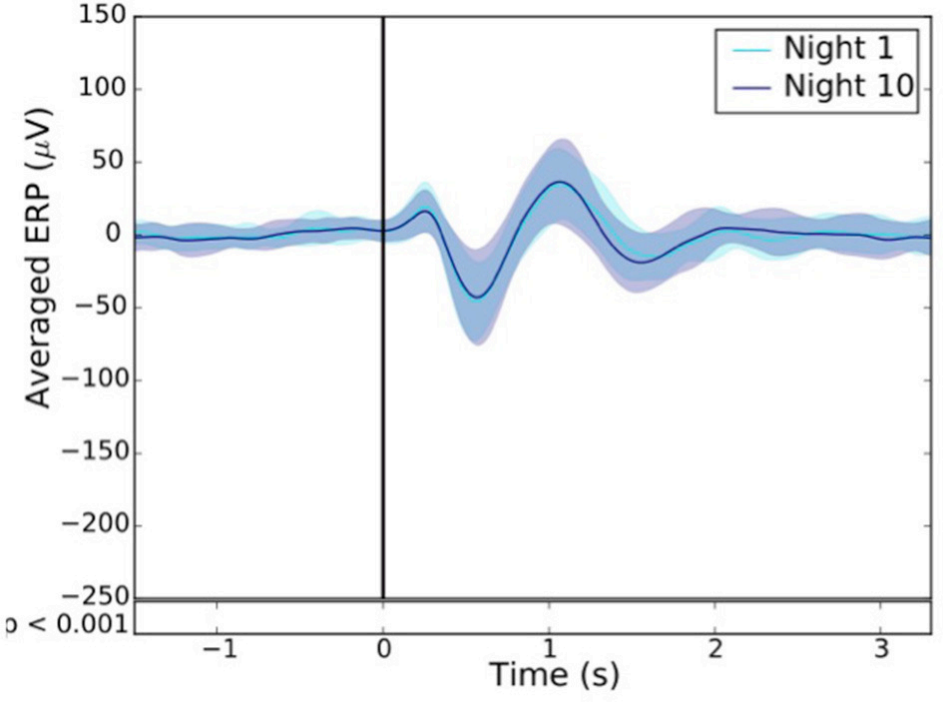
Resulting ERP of the 1st (light blue line) and 10th night (dark blue line) stimuli where the “1st night” and the “10th night” refer to the difference between the stim and the sham of the first and tenth night respectively. Black line indicates the stimuli trigger. Black bars indicate time points where differences between the two conditions were statistically significant (*p* < 0.001).

Across all nights and all stimulations, a total of 10,512 stimulations and 9,872 sham triggers were displayed. The averaged ERP, time-locked to the first (Figure 10A) and the second stimulations (Figure 10B), elicited a greater increase in the amplitude of slow oscillatory activity in the Stimulation condition, as compared to sham, with this effect tapering off after the second oscillation (*p* < 0.001). On average, the power increase in the delta band of the 4 s following the first stimulation was of 43.88% (*p* = 1.20 e-21) as compared to sham triggers (Figure 11). This increase in the delta band was still visible in the 4 s window following the end of the second after the last stimulation with a delta increase of 11.79% (*p* = 3.52 e-5).Finally, no difference was observed on averaged ERP after wearing the device for 10 consecutive nights–i.e., when comparing the impact of the 1st night with stimulation to the 10th night with stimulation (Figure 12).

## Discussion

The present research aimed to assess the performances of the WDD, an ambulatory dry-electrodes EEG device, for auditory closed-loop stimulation of SO during N3 sleep in the home environment. Here, we reported its technical performance from a clinical trial including 20 healthy participants. First, we showed that the device had a good acquisition quality compared to a PSG, with a good ability to detect N3 sleep in real time (specificity: 0.90, sensibility: 0.70) and a precise algorithm for auditory closed-loop stimulation (45 ± 52° reached on average for a 45° targeting). Then, we observed that auditory closed-loop stimulation on a large number of participants in the home environment led to similar results as precedent research have shown in the literature on small samples. Finally, we addressed for the first time the impact of N3 stimulation over 10 nights and showed that the EEG responses to the stimulation after 10 nights were not different from the ones in the first stimulation night.

The experimental procedure following part 1 led to good results in terms of acquisition with good visual identification of sleep patterns (Figure 6), good correlation between signals when using Pearson correlations in 2 s windows (Figure 5) and when comparing the whole night spectrograms (Figure 7). Of note, 4.72% of the WDD recordings were not usable because of bad signal on the two derivations against 1.19% of the PSG recordings. While in clinical practice, considerable time and attention are deployed for the EEG set-up, it is encouraging to observe that subjects were able to use the WDD by themselves to both launch the recording and place the headband. The autonomous placement of the headband by the subjects might have lead to a small offset compared to the optimal position of the electrodes for the comparison between the two devices. This displacement might have contributed to the values dispersion on the Pearson correlation scatter plot (Figure 5).

The detection of N3 sleep, as compared to a PSG gold-standard, led to a specificity of 0.90 and a sensitivity of 0.70 (Table 2), which must be put in perspective with the fact that the inter-scorer variability for sleep stage classification along the AASM rules is about 82% (Younes et al., 2016) and usually under 70% for N3 detection (e.g., 69% in Danker-Hopfe et al., 2009; 67.4% in Rosenberg and Van Hout, 2014). These results were obtained with dry frontal electrodes referred to mastoids whereas the PSG encompassed EEG, EOG and EMG. Indeed, post-processed automatic sleep staging using EEG, EOG and EMG reported performance of 0.92 for specificity and 0.74 for sensitivity (Lajnef et al., 2015). A similar commercial device, the Zeo Wireless using frontal electrodes solely, showed a specificity of 0.62 (Griessenberger et al., 2013) as compared to Somnolyzer, a standard automatic sleep stager (Anderer et al., 2005). Importantly and as opposed to most results in the literature, the WDD algorithms described here run in real-time and are fully embedded on the headband with no outside communication (e.g., Bluetooth, Wi-Fi, etc.) during the night. This imposed significant optimization constraints on all computations performed and drastically oriented the nature of the algorithms used: forest of decision trees rather than deep learning approach. Nonetheless, good performances were reached mainly due to the vertical technological integration of the WDD, which led to a precise optimization of the algorithm.

Auditory closed-loop studies mostly agree that the timing of SO stimulation matters (Cox et al., 2014; Ngo et al., 2015; Weigenand et al., 2016). Therefore, studies were led to improve the stimulation algorithm. In our study, we aimed at delivering auditory stimulation in the ascending phase i.e., 45° of the SO. On average, our algorithm reached 45 ± 52° (Figure 9). To our knowledge, this performance is higher than the phase locked loop (PLL) algorithms previously published. In the initial auditory closed-loop study, the up phase of the stimulation was targeted but the data to assess the precision of the algorithm were not available (Ngo et al., 2013, 2015). In 2014, the PLL algorithm used in the study of Cox and collaborators aimed to target the SO up and down phases (Cox et al., 2014). The performance for the up phase (90°) targeting reached 79 ± 66° on average, indicating a 11° difference from the targeted placement. More recently, a study showed that the stimulation accuracy was improved by stimulating at 50 ± 27°For a targeted placement of 60° in a protocol including sleep naps (Ong et al., 2016). Another recent study achieved a very good performance in elderly participants and reached 329 ± 73° on average for a 340° targeting (Papalambros et al., 2017). Notably, all these studies were led in a sleep lab with an experimental setting requiring the use of complex wiring connected to a computer, and sometimes sleep technicians to initiate the stimulation algorithm when N3 occurred. To our knowledge, only one study used a possible ambulatory set-up where computations were processed on a tablet right next to the bed (Leminen et al., 2017). The performance of the algorithm in this study showed a difference of 18 ± 67°From the desired phase.

The good acquisition performance made possible the ability to stimulate during N3 sleep precisely on the ascending phase of the SO on a large number of participants (90 participants in Part 2). As observed in previous studies including about 10–20 participants (Ngo et al., 2013, 2015; Ong et al., 2016; Leminen et al., 2017; Papalambros et al., 2017), the auditory closed-loop stimulation inspired by Ngo's protocol over our 1,000 nights led to an increase in amplitude during the period immediately following the stimulation. In particular, the power increase of the signal in the delta band (0.4–4 Hz) increased 4 s after a stimulation suggesting a strong local impact. As displayed in Figure 10, the averaged ERPs showed a strong amplification due to stimulation. This is to be understood as a synchronization of brain SO on the stimulation showing a strong local interaction between stimuli and brain activity even during N3 sleep. The increased standard deviation due to stimulation displayed in Figure 11 showed an increase in the amplitude of the SO, which returns to its normal state after ~5 s. In light of our present results, we can not rule out the fact that the ERPs observed were evoked potentials which are not real SO. The analysis of the longitudinal effects of auditory closed-loop stimulation through 10 consecutive nights showed no significant difference as compared to the stimulation of a single night, suggesting that no adaptation mechanism occurs to regulate the impact of the stimulation through the night or with daily stimulation (Figure 12). This observation can be seen as a good thing since it might mean that the brain is not actively compensating for the perturbation in a way that would limit long-term usefulness.

Overall, these results suggest that the auditory stimulation provided by the bone conduction instead of habitual headphones or loudspeakers were similarly able to activate the non-lemniscal pathway and thus trigger slow waves in response to the auditory stimulus (Bellesi et al., 2014). This finding is rather encouraging as it implies that co-sleepers could be individually stimulated with the WDD, which would not have been possible with traditional montage.

In this observational study, data were issued from a “conservative” approach, consisting of choosing stimulation parameters to avoid awaking up the WDD's users as a first objective. Therefore the volume was kept low (40 dB), the time before stimulating set at 15 min, the number of stimulations was moderate during a night (~100 per nights) and stimulations were exclusively triggered during N3 sleep. Moreover, we deliberately tuned our N3 detection algorithms to reach a high specificity at the expense of sensitivity. Indeed, to limit potential awakenings due to sound stimulation in other sleep stages, the WDD was designed to be particularly good at detecting periods identified as different from N3 sleep. In other words, it is crucial that the algorithm does not make mistakes at declaring that a given period is N3 sleep, even at the expense of missing some ambiguous periods. As discussed by Bellesi et al., we believe that the optimization of stimulations parameters such as the targeted sleep stage (N2), the volume intensity, the type of sound, the phase of the stimulation and the number of stimulations (overall or in a single train) could possibly lead to better deep sleep enhancement. Moreover, the age of the subject may change the way the brain responds to auditory stimulation, as the brain and thus the sleep EEG is already different (Papalambros et al., 2017). A personalization approach may thus maximize the effect of auditory stimulation and lead to different results as the one we presented after a single or several stimulation nights.

One limitation of the study is subject attrition due to recording quality (Figure 1). Further, the observational study suffers from the absence of control over the subject's behavior. Subjects bought the WDD and used it on a voluntarily basis. There was no proper recruitment or screening and we had little information about their profiles and habits. In particular, their sleep habits, disease, drugs consumption, etc., were not verified. It must also be noted that a recruitment bias is possible since subjects who keep using the WDD after numerous nights may also be the subjects whose sleep was responsive to the stimulation. These constraints were inherent to any observational ecological study, and may alter some findings about the global population of users. Also, while it is usually considered that females have a higher rate of sleep issues, many studies only include males so as to avoid hormonal bias related to menstrual cycles. Therefore, we do not think that the disbalance in terms of sex ratio is a real bias for the data analysis and will change the comparison with previous published litterature.

Overall, since we restricted our analysis to the quantification local physiological impacts of the stimulation, we do not believe that any of these biases significantly impacted the results presented here. Nonetheless a systematic study covering the longitudinal effects of stimulation as well as studies including more women is needed to confirm our results.

Regarding our results, it can be emphasized that the methods used did not gave the possibility to determine whether the evoked responses observed after stimulation were proper SO or auditory cortex response only. More complex methods and set-up associating MEG or full cap EEG source as well as the device would have been a way to answer to that question. Also, the subjective and objective effect of repeated auditory stimulation on sleepiness, cognition, immunity and overall health was not assessed. This would be of interest for a better understanding of SWS boosting in both healthy and young as well as unhealthy and elderly subjects. Also, in the present paper, we did not address and analyze the interest of precise timing and how it can potentially impact the EEG response as well as the whole cognition. As suggested by several papers, timing seems indeed to “matter” (Weigenand et al., 2016) with the majority of the publications targeting the ascending or up state of the SO after the second paper of Ngo & al on auditory closed-loop stimulation (Ngo et al., 2015). In the TMR application field, timing seems also to matter since memory can be either boosted or blocked depending on the timing of application of correct of conflicting feedbacks (Schreiner et al., 2015). Now that the recording quality and the stimulation precision of the algorithm are assessed, further papers should address this question of timing.

To summarize, this study showed that the WDD, a fully integrated dry-electrode commercial wearable, could monitor sleep on frontal EEG derivations with a good acquisition quality compared to gold-standard PSG devices. The specificity and sensibility to detect N3 sleep as well as its stimulation accuracy were above the performances found in the literature. Its use in the home environment has resulted in an unprecedented number of nights as compared to what we found in the literature on this topic (Ngo et al., 2013, 2015; Cox et al., 2014; Leminen et al., 2017; Papalambros et al., 2017). We were able to replicate previous studies on EEG response to auditory closed-loop stimulations and showed for the first time that these stimulations over 10 nights did not reduce nor potentiate the EEG responses of a single stimulation night.

Overall, given its performance and its ease of use, the WDD may be an excellent way to go further into the analysis of N3 sleep stimulations, including targeting memory reactivation on larger populations than in anterior works. More broadly, it provides new opportunities on the assessment of sleep EEG biomarkers by possibly being a substitute of PSG in “outside of the lab” longitudinal cohort studies.

## Author contributions

Study concept and design: ED, MG, PA, DL, and MC. Data acquisition: ED and PA. Data analysis: MG, SC, CP, DD, VT, and ED. Data interpretation: ED, MG, PA, MC, and DL. Writing the manuscript for content: ED and MG. Revising the manuscript: DL, MC, MG, and PA.

### Conflict of interest statement

ED, SC, CP, VT, DD, PA, and MG are employees of Rythm. The other authors declare that the research was conducted in the absence of any commercial or financial relationships that could be construed as a potential conflict of interest.

## Supplementary material

Figure S1 Individual multitaper EEG spectrogram of a full sleep night from the WDD **(Top)** and on the PSG **(Bottom)** recordings.

Figure S2 Individual hypnograms scored by the sleep expert. Stimulation triggers are shown in red.

Figure S3 Individual polar plots. The targeted phase was 45° which represents the middle of the ascending slope. 90° corresponds to the peak of the up state, 270 degrees to the trough of the down state.

## References

[B1] Adali T. Haykin S. (2010). Adaptive Signal Processing: Next-Generation Solutions, Vol. 55 JohnWiley & Sons.

[B2] Anderer P. Gruber G. Parapatics S. Woertz M. Miazhynskaia T. Klösch G.. (2005). An E-Health solution for automatic sleep classification according to Rechtschaffen and Kales: validation study of the somnolyzer 24 x 7 utilizing the siesta database. Neuropsychobiology 51, 115–133. 10.1159/000085205 15838184

[B3] Bayer L. Constantinescu I. Perrig S. Vienne J. Vidal P. P. Mühlethaler M.. (2011). Rocking synchronizes brain waves during a short nap. Curr. Biol. 21, R461–R462. 10.1016/j.cub.2011.05.012 21683897

[B4] Bellesi M. Riedner B. A. Garcia-Molina G. N. Cirelli C. Tononi G. (2014). Enhancement of sleep slow waves: underlying mechanisms and practical consequences. Front. Syst. Neurosci. 8:208 10.3389/fnsys.2014.00208 25389394 PMC4211398

[B5] Besedovsky L. Lange T. Born J. (2012). Sleep and immune function. Pflugers Arch. Eur. J. Physiol. 463, 121–137. 10.1007/s00424-011-1044-0 22071480 PMC3256323

[B6] Besedovsky L. Ngo H. V. V. Dimitrov S. Gassenmaier C. Lehmann R. Born J. (2017). Auditory closed-loop stimulation of EEG slow oscillations strengthens sleep and signs of its immune-supportive function. Nat. Commun. 8:1984. 10.1038/s41467-017-02170-3 29215045 PMC5719447

[B7] Born J. (2010). Slow-wave sleep and the consolidation of long-term memory. World J. Biol. Psychiatry 11(Suppl. 1), 16–21. 10.3109/15622971003637637 20509828

[B8] Cox R. Korjoukov I. De Boer M. Talamini L. M. (2014). Sound asleep: processing and retention of slow oscillation phase-targeted stimuli. PLoS ONE 9:e101567. 10.1371/journal.pone.0101567 24999803 PMC4084884

[B9] Danker-Hopfe H. Anderer P. Zeitlhofer J. Boeck M. Dorn H. Gruber G.. (2009). Interrater reliability for sleep scoring according to the Rechtschaffen & Kales and the new AASM standard. J. Sleep Res. 18, 74–84. 10.1111/j.1365-2869.2008.00700.x 19250176

[B10] Griessenberger H. Heib D. P. J. Kunz A. B. Hoedlmoser K. Schabus M. (2013). Assessment of a wireless headband for automatic sleep scoring. Sleep Breath. 17, 747–752. 10.1007/s11325-012-0757-4 22996794 PMC3655221

[B11] Gumenyuk V. Roth T. Korzyukov O. Jefferson C. Bowyer S. Drake C. L. (2011). Habitual short sleep impacts frontal switch mechanism in attention to novelty. Sleep 34, 1659–1670. 10.5665/sleep.1430 22131603 PMC3208843

[B12] Honma M. Plass J. Brang D. Florczak S. M. Grabowecky M. Paller K. A. (2016). Sleeping on the rubber-hand illusion: memory reactivation during sleep facilitates multisensory recalibration. Neurosci. Conscious. 2016:niw020. 10.1093/nc/niw020 28184322 PMC5294922

[B13] Iber C. Ancoli-Israel S. Chesson A. Quan S. (2007). The AASM Manual for the Scoring of Sleep and Associates Events. Rules, Terminology and Technical Specifications.

[B14] Irish L. A. Kline C. E. Gunn H. E. Buysse D. J. Hall M. H. (2015). The role of sleep hygiene in promoting public health: a review of empirical evidence. Sleep Med. Rev. 22, 23–36. 10.1016/j.smrv.2014.10.001 25454674 PMC4400203

[B15] Kudesia R. S. Bianchi M. T. (2012). Decreased nocturnal awakenings in young adults performing bikram yoga: a low-constraint home sleep monitoring study. ISRN Neurol. 2012:153745. 10.5402/2012/153745 22577578 PMC3345216

[B16] Lajnef T. Chaibi S. Ruby P. Aguera P. E. Eichenlaub J. B. Samet M.. (2015). Learning machines and sleeping brains: automatic sleep stage classification using decision-tree multi-class support vector machines. J. Neurosci. Methods 250, 94–105. 10.1016/j.jneumeth.2015.01.022 25629798

[B17] Leminen M. M. Virkkala J. Saure E. Paajanen T. Zee P. C. Santostasi G.. (2017). Enhanced memory consolidation via automatic sound stimulation during non-REM sleep. Sleep 40:zsx003. 10.1093/sleep/zsx003 28364428 PMC5806588

[B18] Lentz M. J. Landis C. A. Rothermel J. Shaver J. L. (1999). Effects of selective slow wave sleep disruption on musculoskeletal pain and fatigue in middle aged women. J. Rheumatol. 26, 1586–1592. 10405949

[B19] Marshall L. Helgadóttir H. Mölle M. Born J. (2006). Boosting slow oscillations during sleep potentiates memory. Nature 444, 610–613. 10.1038/nature05278 17086200

[B20] Mathias S. Wetter T. C. Steiger A. Lancel M. (2001). The GABA uptake inhibitor tiagabine promotes slow wave sleep in normal elderly subjects. Neurobiol. Aging 22, 247–253. 10.1016/S0197-4580(00)00232-3 11182474

[B21] Mihajlovic V. Grundlehner B. Vullers R. Penders J. (2015). Wearable, wireless EEG solutions in daily life applications: what are we missing? IEEE J. Biomed. Heal. Informatics 19, 6–21. 10.1109/JBHI.2014.2328317 25486653

[B22] Ngo H. V. V. Miedema A. Faude I. Martinetz T. Mo M. (2015). Driving sleep slow oscillations by auditory closed-loop stimulation — a self-limiting Process. J. Neurosci. 35, 6630–6638. 10.1523/JNEUROSCI.3133-14.2015 25926443 PMC4412888

[B23] Ngo H. V. V. Martinetz T. Born J. Mölle M. (2013). Auditory closed-loop stimulation of the sleep slow oscillation enhances memory. Neuron 78, 545–553. 10.1016/j.neuron.2013.03.006 23583623

[B24] Ong J. L. Lo J. C. Chee N. I. Santostasi G. Paller K. A. Zee P. C.. (2016) Effects of phase-locked acoustic stimulation during a nap on EEG spectra declarative memory consolidation. Sleep Med. 20, 88–97. 10.1016/j.sleep.2015.10.016 27318231

[B25] Oudiette D. Santostasi G. Paller K. A. (2013). Reinforcing rhythms in the sleeping brain with a computerized metronome. Neuron 78, 413–415. 10.1016/j.neuron.2013.04.032 23664610 PMC3679648

[B26] Papalambros N. A. Santostasi G. Malkani R. G. Braun R. Weintraub S. Paller K. A.. (2017). Acoustic enhancement of sleep slow oscillations and concomitant memory improvement in older Adults. Front. Hum. Neurosci. 11:109. 10.3389/fnhum.2017.00109 28337134 PMC5340797

[B27] Rosenberg R. S. Van Hout S. (2014). The american academy of sleep medicine inter-scorer reliability program: respiratory events. J. Clin. Sleep Med. 10, 447–454. 10.5664/jcsm.3630 24733993 PMC3960390

[B28] Sauvet F. Bougard C. Coroenne M. Lely L. Van Beers P. Elbaz M.. (2014). In-flight automatic detection of vigilance states using a single EEG channel. IEEE Trans. Biomed. Eng. 61, 2840–2847. 10.1109/TBME.2014.2331189 24967979

[B29] Schreiner T. Lehmann M. Rasch B. (2015). Auditory feedback blocks memory benefits of cueing during sleep. Nat. Commun. 6:8729. 10.1038/ncomms9729 26507814 PMC4640077

[B30] Scullin M. K. (2012). Sleep, memory, and aging: the link between slow-wave sleep and episodic memory changes from younger to older adults. Psychol. Aging 28, 105–114. 10.1037/a0028830 22708533 PMC3532961

[B31] Shambroom J. R. Fábregas S. E. Johnstone J. (2012). Validation of an automated wireless system to monitor sleep in healthy adults. J. Sleep Res. 21, 221–230. 10.1111/j.1365-2869.2011.00944.x 21859438

[B32] Tonetti L. Cellini N. de Zambotti M. Fabbri M. Martoni M. Fábregas S. E.. (2013). Polysomnographic validation of a wireless dry headband technology for sleep monitoring in healthy young adults. Physiol. Behav. 118, 185–188. 10.1016/j.physbeh.2013.05.036 23714587

[B33] Tononi G. Cirelli C. (2003). Sleep and synaptic homeostasis: a hypothesis. Brain Res. Bull. 62, 143–150. 10.1016/j.brainresbull.2003.09.004 14638388

[B34] Van Cauter E. Plat L. Scharf M. B. Leproult R. Cespedes S. L'Hermite-Balériaux M.. (1997). Simultaneous stimulation of slow-wave sleep and growth hormone secretion by gamma-hydroxybutyrate in normal young men. J. Clin. Invest. 100, 745–753. 9239423 10.1172/JCI119587 PMC508244

[B35] Van De Water A. T. M. Holmes A. Hurley D. A. (2011). Objective measurements of sleep for non-laboratory settings as alternatives to polysomnography - a systematic review. J. Sleep Res. 20, 183–200. 10.1111/j.1365-2869.2009.00814.x 20374444

[B36] Varin C. Rancillac A. Geoffroy H. Arthaud S. Fort P. Gallopin T. (2015). Glucose induces slow-wave sleep by exciting the sleep-promoting neurons in the ventrolateral preoptic nucleus: a new link between sleep and metabolism. J. Neurosci. 35, 9900–9911. 10.1523/JNEUROSCI.0609-15.2015 26156991 PMC6605416

[B37] Walsh J. K. Zammit G. Schweitzer P. K. Ondrasik J. Roth T. (2006). Tiagabine enhances slow wave sleep and sleep maintenance in primary insomnia. Sleep Med. 7, 155–161. 10.1016/j.sleep.2005.05.004 16260179

[B38] Weigenand A. Mölle M. Werner F. Martinetz T. Marshall L. (2016). Timing matters: open-loop stimulation does not improve overnight consolidation of word pairs in humans. Eur. J. Neurosci. 44, 2357–2368. 10.1111/ejn.13334 27422437 PMC5113809

[B39] Xie L. Kang H. Xu Q. Chen M. J. Liao Y. Thiyagarajan M.. (2013). Sleep drives metabolite clearance from the adult brain. Science 342, 373–377. 10.1126/science.1241224 24136970 PMC3880190

[B40] Younes M. Raneri J. Hanly P. (2016). Staging sleep in polysomnograms: analysis of inter-scorer variability. J. Clin. Sleep Med. 12, 885–894. 10.5664/jcsm.5894 27070243 PMC4877322

