# Supplementary figures and images for: Performance of an Ambulatory Dry-EEG Device for Auditory Closed-Loop Stimulation of Sleep Slow Oscillations in the Home Environment
Source: Front Hum Neurosci. 2018 Mar 8;12:88. doi: 10.3389/fnhum.2018.00088 (PMC5853451; doi:10.3389/fnhum.2018.00088)

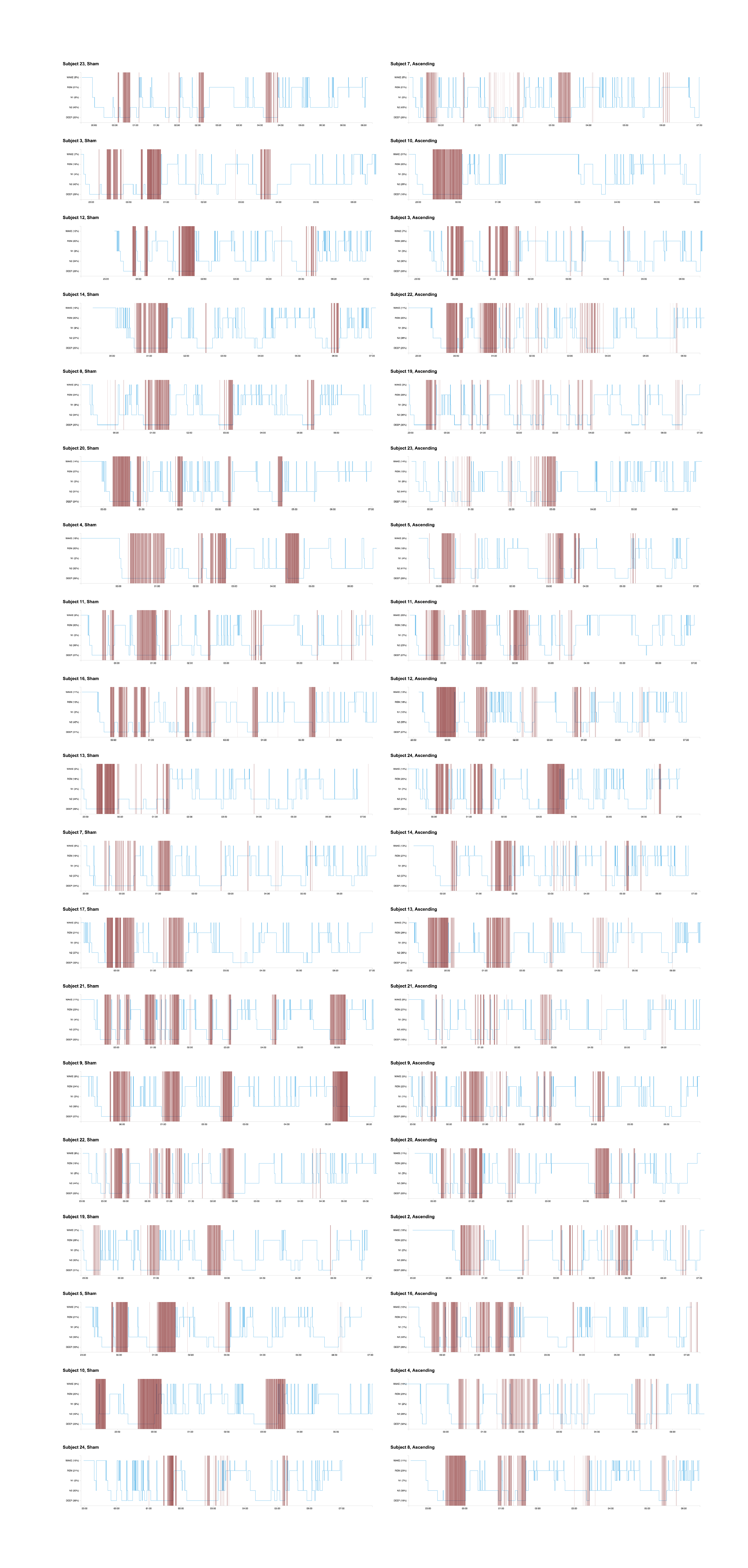

Supplement: Figure S2 — Individual hypnograms scored by the sleep expert. Stimulation triggers are shown in red. [file Image2.JPEG]

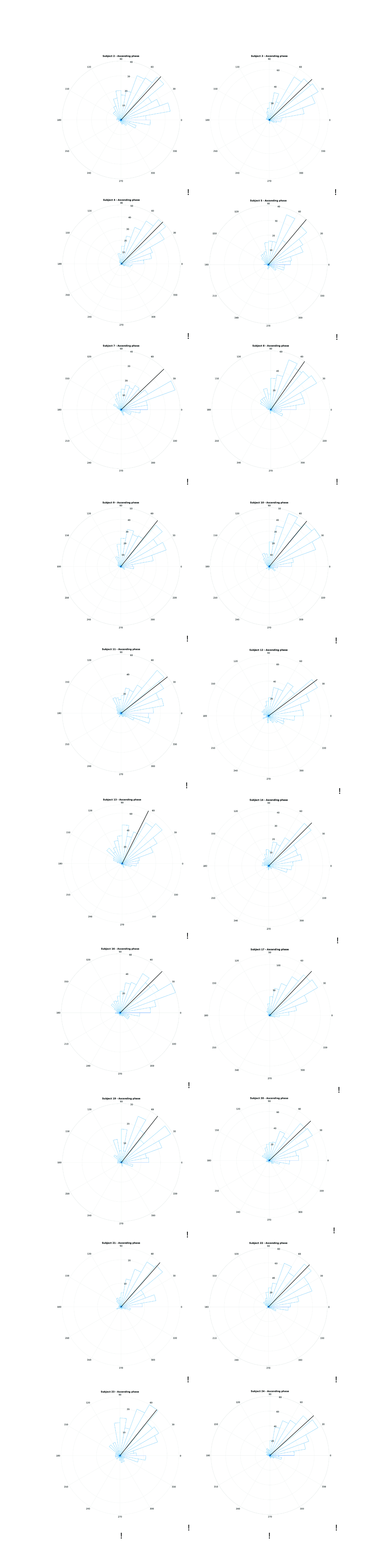

Supplement: Figure S3 — Individual polar plots. The targeted phase was 45° which represents the middle of the ascending slope. 90° corresponds to the peak of the up state, 270 degrees to the trough of the down state. [file Image3.jpeg]
